# Krüppel-Like Factor 4 Overexpression Initiates a Mesenchymal-to-Epithelial Transition and Redifferentiation of Human Pancreatic Cells following Expansion in Long Term Adherent Culture

**DOI:** 10.1371/journal.pone.0140352

**Published:** 2015-10-12

**Authors:** Kenneth R. Muir, Maria João Lima, Hilary M. Docherty, Neil W. A. McGowan, Shareen Forbes, Yves Heremans, Stuart J. Forbes, Harry Heimberg, John Casey, Kevin Docherty

**Affiliations:** 1 School of Medical Sciences, University of Aberdeen, Institute of Medical Sciences, Foresterhill, Aberdeen, United Kingdom; 2 Department of Surgery, University of Edinburgh, Edinburgh Royal Infirmary, Edinburgh, United Kingdom; 3 Endocrinology Unit, University/BHF Centre for Cardiovascular Science, Queen’s Medical Research Institute, University of Edinburgh, Edinburgh, United Kingdom; 4 Diabetes Research Center, Vrije Universiteit Brussel, Brussels, Belgium; 5 MRC Centre for Regenerative Medicine, SCRM Building, The University of Edinburgh, Edinburgh, United Kingdom; University of Alabama at Birmingham, UNITED STATES

## Abstract

A replenishable source of insulin-producing cells has the potential to cure type 1 diabetes. Attempts to culture and expand pancreatic β-cells *in vitro* have resulted in their transition from insulin-producing epithelial cells to mesenchymal stromal cells (MSCs) with high proliferative capacity but devoid of any hormone production. The aim of this study was to determine whether the transcription factor Krüppel-like factor 4 (KLF4), could induce a mesenchymal-to-epithelial transition (MET) of the cultured cells. Islet-enriched pancreatic cells, allowed to dedifferentiate and expand in adherent cell culture, were transduced with an adenovirus containing KLF4 (Ad-Klf4). Cells were subsequently analysed for changes in cell morphology by light microscopy, and for the presence of epithelial and pancreatic markers by immunocytochemistry and quantitative RT/PCR. Infection with Ad-Klf4 resulted in morphological changes, down-regulation of mesenchymal markers, and re-expression of both epithelial and pancreatic cell markers including insulin and transcription factors specific to β-cells. This effect was further enhanced by culturing cells in suspension. However, the effects of Ad-KLf4 were transient and this was shown to be due to increased apoptosis in Klf4-expressing cells. Klf4 has been recently identified as a pioneer factor with the ability to modulate the structure of chromatin and enhance reprogramming/transdifferentiation. Our results show that Klf4 may have a role in the redifferentiation of expanded pancreatic cells in culture, but before this can be achieved the off-target effects that result in increased apoptosis would need to be overcome.

## Introduction

Transplantation of islets holds great promise as a cure for type 1 diabetes. The introduction of the Edmonton protocol in 2000 demonstrated that human donor islet transplantation can lead to a significant decrease in exogenous insulin requirements and even temporary insulin independence along with reduction of severe hypoglycaemia [1]. Islet cell transplantation is limited by the availability of donor tissue; therefore an alternative replenishable source of β-cells is required. Using adult human β-cells as a starting population and expanding them *in vitro* would seem like an obvious solution, but is one that has been met with little progress despite considerable effort [2].

Isolated human islets of Langerhans can be maintained as functional units in suspension culture for many months without proliferation [3,4]. However, when human islets are placed in adherent culture conditions, fibroblast-like cells migrate out from the islet foci [5]. These cells can proliferate and form a monolayer that can be grown to passage 20 and beyond. A similar scenario occurs when the islets are dispersed and plated as single cells [6]. Formation of the fibroblast-like monolayer is accompanied by loss of epithelial markers, acquisition of mesenchymal markers and loss of hormone secretion from the islets including insulin and other hormones. The fibroblast-like cells express cell surface markers (CD90, CD107 and CD73) of mesenchymal stromal cells (MSC) and can, in keeping with the properties of MSCs, be induced to redifferentiate towards osteoblast, chondrocyte and adipocyte lineages. There is some controversy concerning the origins of the MSCs that occur when islets are placed in culture. Genetic lineage tracing studies in mice showed that β-cells dedifferentiated in culture but failed to proliferate and were eliminated from the culture [7–9]. However, genetically traced cultured human β-cells dedifferentiate and replicate [6,10,11]. It is likely that the MSC population arises from dedifferentiated epithelial cells via a process of EMT as well as from passenger stromal cells.

If this process can be reversed, i.e. by inducing a mesenchymal-to-epithelial transition (MET) there is potential to generate clinically meaningful numbers of β-cells [12]. Some progress has been made. Thus when human islet-derived MSCs are transferred from serum-containing to serum-free medium, the cells form epithelial-like clusters and re-express low levels of endocrine hormones [5,13]. This effect can be enhanced by addition of soluble factors or by targeting components of the EMT signalling pathway [14–16].

It is of relevance that MET [17,18] may be an early and essential process in the generation of induced pluripotent stem cells (iPSCs) from murine fibroblasts using the transcription factor cocktail Oct4, Sox2, Klf4 and c-Myc [19]. Krüppel-like factor 4 (KLF4), a multi-zinc finger SP1-like transcription factor, appears fundamental to this process, as when overexpressed in the absence of the other transcription factors, epithelial markers were up-regulated significantly [18]. Furthermore, KLF4 was shown to bind to the E-cadherin promoter [20,21] and to act as a transcriptional repressor of genes critical for EMT, including SLUG and JNK1 [22].

We hypothesised that KLF4 may also play a similar role in promoting a MET in dedifferentiated pancreatic cells, and if these cells retained epigenetic memory of their origins, as suggested by other studies, it would allow preferential lineage-specific differentiation. If feasible, this strategy would have the potential to produce a replenishable supply of β-cells through targeting pathways required for MET, whilst bypassing pluripotency and its associated risks. Here we demonstrate that KLF4 can initiate a transient MET in MSCs derived from islet-enriched pancreatic cells, as evidenced by up-regulation of epithelial markers and down-regulation of mesenchymal markers. However, KLF4 also promoted cell death via apoptosis. This suggests that before transcription factor mediated reversal of MET can play a role in cell therapy these off- target effects of KLF4 would need to be addressed.

## Materials and Methods

### Culture of human islet- enriched pancreatic fractions

All human tissue was procured with written informed consent from the donor or next of kin and with ethical approval from the North of Scotland Research Ethics Committee (REC reference number 10/S0802/12). Human islets were isolated from brain-dead adult donor pancreata at the Scottish Islet Isolation Laboratory, Edinburgh, UK, under GMP conditions. Islet-enriched fractions were plated at a density of 3 x 10^5^ clusters on 75 cm^2^ tissue culture flasks (Greiner, Stonehouse, UK) and cultured in serum-containing medium (SCM) prepared using RPMI 1640 (Gibco, Life Technologies, Paisley, UK) supplemented with 10% FBS, 10 mM HEPES, 1 mM sodium pyruvate (all from Gibco), and 75 μM β-mercaptoethanol (Sigma Aldrich, Dorset, UK). Cells were passaged every 5–7 days using trypsin (0.05%) and EDTA (0.02%, Gibco). Serum-free medium (SFM) was prepared with RPMI supplemented with 1% BSA and 10 μg/ml insulin, 5.5 μg/ml transferrin and 6.7 ng/ml sodium selenite.

### Viral vectors

Adenoviral vectors pAd-Klf4 and pAd-EGFP were obtained from Addgene. Adenoviral-mediated transduction was performed in SFM at a multiplicity of infection of 25. Reprogramming using Ad-Pdx1, Ad-MafA, Ad-Ngn3 and Ad-Pax4 (4TFs) was as previously described [11].

### Genetic Lineage Tracing

Genetic lineage tracing was performed as previously described [11].

### Fluorescence activated cell sorting

Cells for sorting were incubated in StemPro® Accutase® (Life Technologies, Paisley UK) for 10 min followed by pipetting to break up clusters. Cells were then passed through a 70 μm cell strainer and dsRed positive cells sorted on a BD Influx^TM^ Cell Sorter using a phycoerythrin 593/40 filter. Collected dsRed- positive cell fractions were then expanded in adherent culture for a further 8 weeks prior to further experiments.

### Differentiation towards adipocyte and osteocyte lineages

Islet-derived dsRed sorted cells were seeded at a density of 2x10^4^ cells on 22x22 mm coverslips. The cells were cultured in the presence of StemPro Osteogenesis differentiation medium (Life Technologies) for 20 days or StemPro Adipogenesis medium (Life Technologies) for 10 days.

### Quantitative RT/PCR

Quantitative RT-PCR was performed as previously described [11]. Samples were run in triplicate and normalised to glyceraldehyde 3-phosphate dehydrogenase (GAPDH). Data were analysed using the 2^–ΔΔCT^ method. Statistical analysis was performed using GraphPad Prism software and the Student *t* test or one-way/two-way ANOVA, followed by the Dunnett post hoc test, were used as appropriate. TaqMan primers sequences are shown in Table 1.

**Table 1:** List of Taqman® gene expression primers.

Gene	Assay Identification
CDH1	Hs01023894_m1
EPCAM	Hs00901885_m1
VIM	Hs00185584_m1
SNAI2	Hs00950344_m1
ZEB1	Hs00232783_m1
ACTA2	Hs00909449_m1
GAPDH	Hs99999905_m1
RPL13A	Hs04194366_g1
INS	Hs00355773_m1
GCG	Hs00174967_m1
SST	Hs001174949_m1
AMY2B	Hs00949916_m1
KRT19	Hs00761767_s1
PDX1	Hs00236830_m1
OCT4	Hs04260367_gH
SOX2	Hs01053049_s1
NANOG	Hs04260366_g1
KLF4 (mouse)	Mm00516105_g1

### Immunocytochemistry

Cells were cultured on 22 x 22 mm or 13 mm round glass coverslips. Immunocytochemistry was performed as previously described [11,23]. Fluorescent Images were captured using a Zeiss Axio Imager.M2 and collated with AxioVision software. Antibodies used are shown in Table 2.

**Table 2:** Primary antibodies used in immunofluorescence.

Antigen	Antibody host	Source	Dilution used
E Cadherin	Mouse	Becton Dickinson	1:200
Vimentin	Rabbit	Dako	1:200
Vimentin	Mouse	Proteintech	1:200
Amylase	Rabbit	Sigma	1:100
KLF4	Rabbit	Millipore	1:300
C-peptide	Mouse	Cell Signalling	1:1000
Cleaved Caspase-3	Rabbit	NE biolabs	1:200
Osteocalcin	Mouse	Abcam	1:50

### TUNEL assay

Cells were seeded on 22x22 mm coverslips and after 24 h infected with Ad-KLF4. The anti-proliferative agent, paclitaxel was used as a positive control to induce apoptosis. A terminal deoxynucleotidyl transferase mediated dUTP nick-end labelling (TUNEL) assay was performed using the ApopTag Fluorescein Direct In Situ Apoptosis Detection kit (Millipore, Watford, UK) according to the manufacturer’s instructions. Cell counts were performed over 10 randomly selected fields over 2 slides with at least 700 nuclei per slide. TUNEL positive cells were identified using the FITC channel on a Zeiss Axio Imager.M2 fluorescence microscope.

## Results

### Freshly isolated islet-enriched pancreatic cells undergo EMT in adherent cell culture

**Fig 1:**
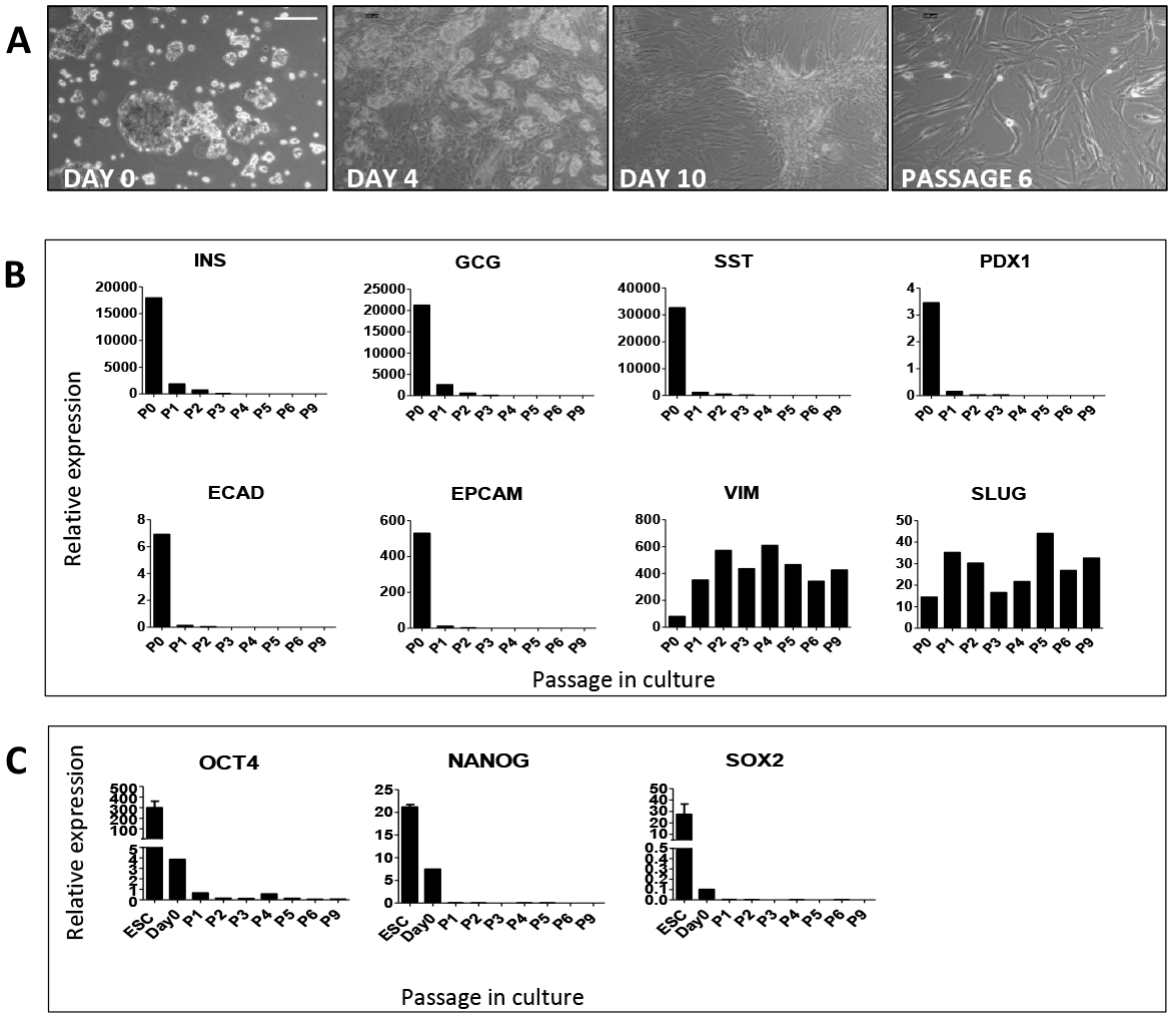
Islet enriched pancreatic cells form fibroblast-like monolayers and dedifferentiate in adherent cell culture. A: Phase contrast images taken in culture from day 0 to passage 6. QRT/PCR analysis of endocrine, epithelial, mesenchymal (B) and pluripotency markers (C) in cells harvested from passage 1 to passage 9 in tissue culture. Data are expressed relative to glyceraldehyde 3-phosphate dehydrogenase. ESC are human embryonic stem cells.

When plated in plastic tissue culture dishes the islet clusters attached to the dish. Within 24 h fibroblast-like cells started to migrate out of the cluster, forming a proliferative monolayer that spread throughout the culture dish (Fig 1A). This monolayer could be repeatedly passaged. At passage 6 (approximately 4 weeks), the cell population resembled a monolayer of mesenchymal stromal cells (Fig 1A). In keeping with previous studies [5,24] there was a rapid decrease in expression of epithelial markers (insulin, glucagon, somatostatin, PDX1, E-cadherin and Ep-cam with a concomitant increase in expression of the mesenchymal markers vimentin and SNAI2 (SLUG) (Fig 1B). We have previously shown that these fibroblast-like cells express surface antigens that are characteristic of mesenchymal stromal cells (MSCs), and in keeping with the properties of MSCs can be differentiated into adipocytes, osteoblasts and chondrocytes [11]. A weak expression (relative to ES cells) of pluripotency markers (OCT4, SOX2 and NANOG) was detected in the newly plated islets; however this was rapidly lost, and there was no transient increase around passage 5 (Fig 1C) as reported by others [25]. We have shown previously [11] that these MSC-like cells arise from dedifferentiation via a process of epithelial to mesenchymal transition of pancreatic endocrine and exocrine cells as well as from expansion of endogenous stromal cells.

### Klf-4 overexpression induces a MET and redifferentiation towards pancreatic cell types

**Fig 2:**
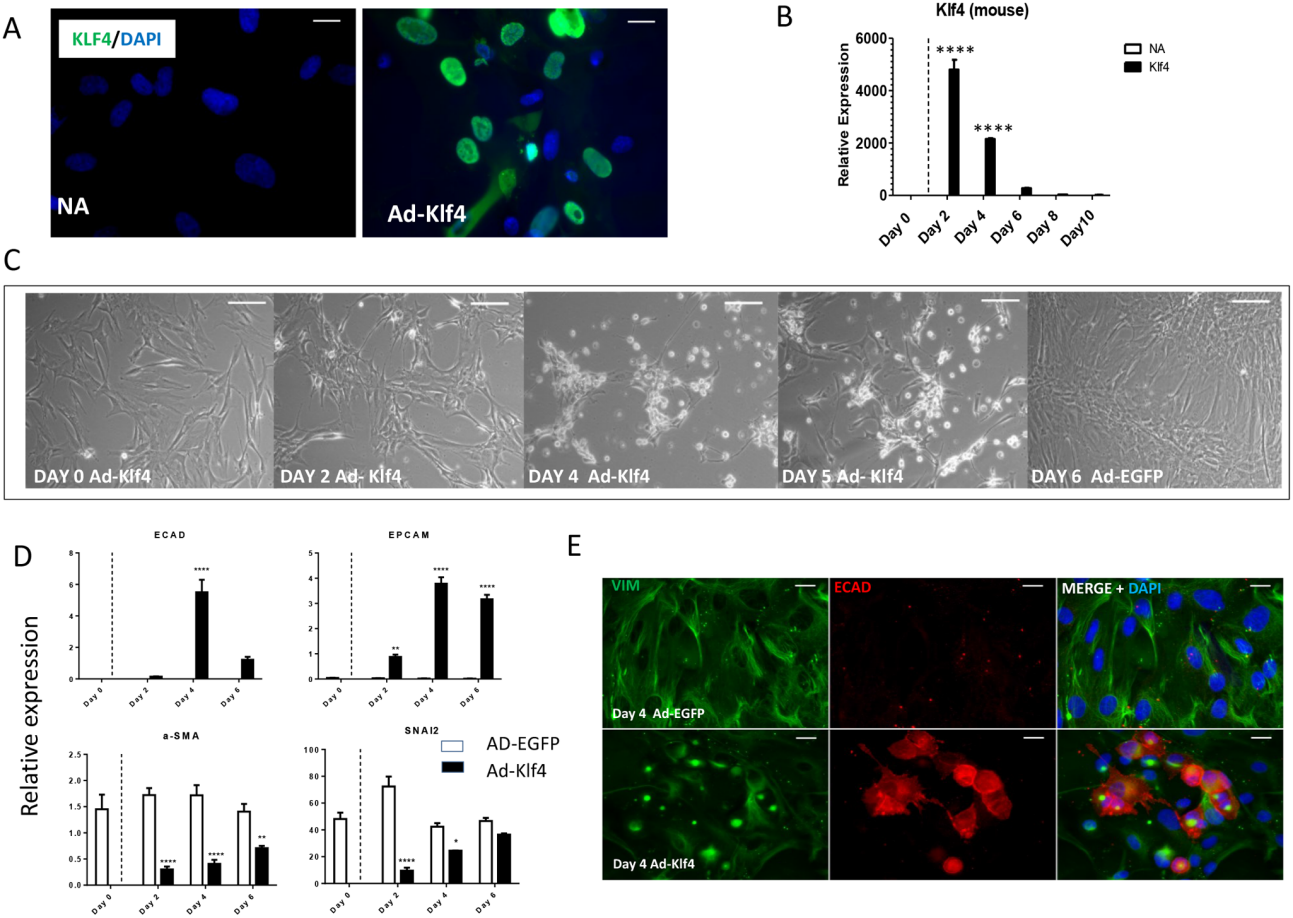
KLF4 overexpression induces morphological change with up-regulation of epithelial markers and down-regulation of mesenchymal markers. Islet enriched pancreatic cell clusters were cultured in RPMI with 10% FBS and allowed to adhere and expand. (A) At passage 6 cells were transduced with Ad-Klf4. The left panel shows untreated cells (NA) and the right panel cells treated with Ad-KLf4. Cells were stained with DAPI (nuclei) and anti-KLF4 (green). (B) QRT/PCR analysis of exogenous (mouse) Klf4 expression up to day 10 in transduced (klf4) and untreated (NA) cells. The dotted line represents the time of adenoviral transduction. Data are expressed relative to glyceraldehyde 3-phosphate dehydrogenase and represent mean ± SEM (n = 3). (C) Phase-contrast images showing morphological changes at time points post infection with Ad-Klf4 or Ad-EGFP. (D) Cells were harvested at time points for gene expression by QRT/PCR. Data were expressed relative to glyceraldehyde-3-phosphate dehydrogenase (n = 3). A two-way ANOVA was performed with Bonferroni post hoc test comparing treatment groups with Ad-EGFP. For all analyses, *P < 0.05 **P < 0.01 ***P < 0.001. (E) Immunocytochemical staining of the epithelial marker E-cadherin and the mesenchymal marker vimentin at day 4 post transduction with Ad-Klf4 versus control. Nuclei were counterstained with DAPI. Scale bar = 20μm.

There was no detectable endogenous KLF4 in untreated cells (Fig 2A). Ad-Klf4 was efficiently taken up by 71.3% ± 15.1% of the islet- derived MSCs (Fig 2A) with rapid expression of the exogenous mouse Klf4 peaking at day 2, followed by a subsequent fall to undetectable levels by day 8 (Fig 2B). Following Ad-Klf4 transduction, the islet-derived MSCs underwent significant morphological changes with aggregation and many cells transitioning towards a more rounded epithelial form (Fig 2C). This was not seen in Ad-EGF treated control cells. In Ad-Klf4, but not Ad-EGFP, treated cells expression of the epithelial markers E-cadherin (ECAD) and epithelial cell adhesion molecule (Ep-CAM) was rapidly up-regulated to significant levels peaking at day 4, with a subsequent decrease towards day 6 (Fig 2D). Widespread E-cadherin staining was detected (38.3% ± 5.5%) in Ad-Klf4 treated cells, but not in Ad-EGFP, transduced cells, with positive cells displaying a more epithelial morphology (Fig 2E). Conversely, expression of the mesenchymal markers α-SMA, and the transcriptional repressor SNAI2 was down-regulated significantly at day 2 after transduction, but increased towards baseline levels upon further culture (Fig 2D). This was accompanied by perinuclear relocation of vimentin in cells staining positive for E-cadherin (Fig 2E).

**Fig 3:**
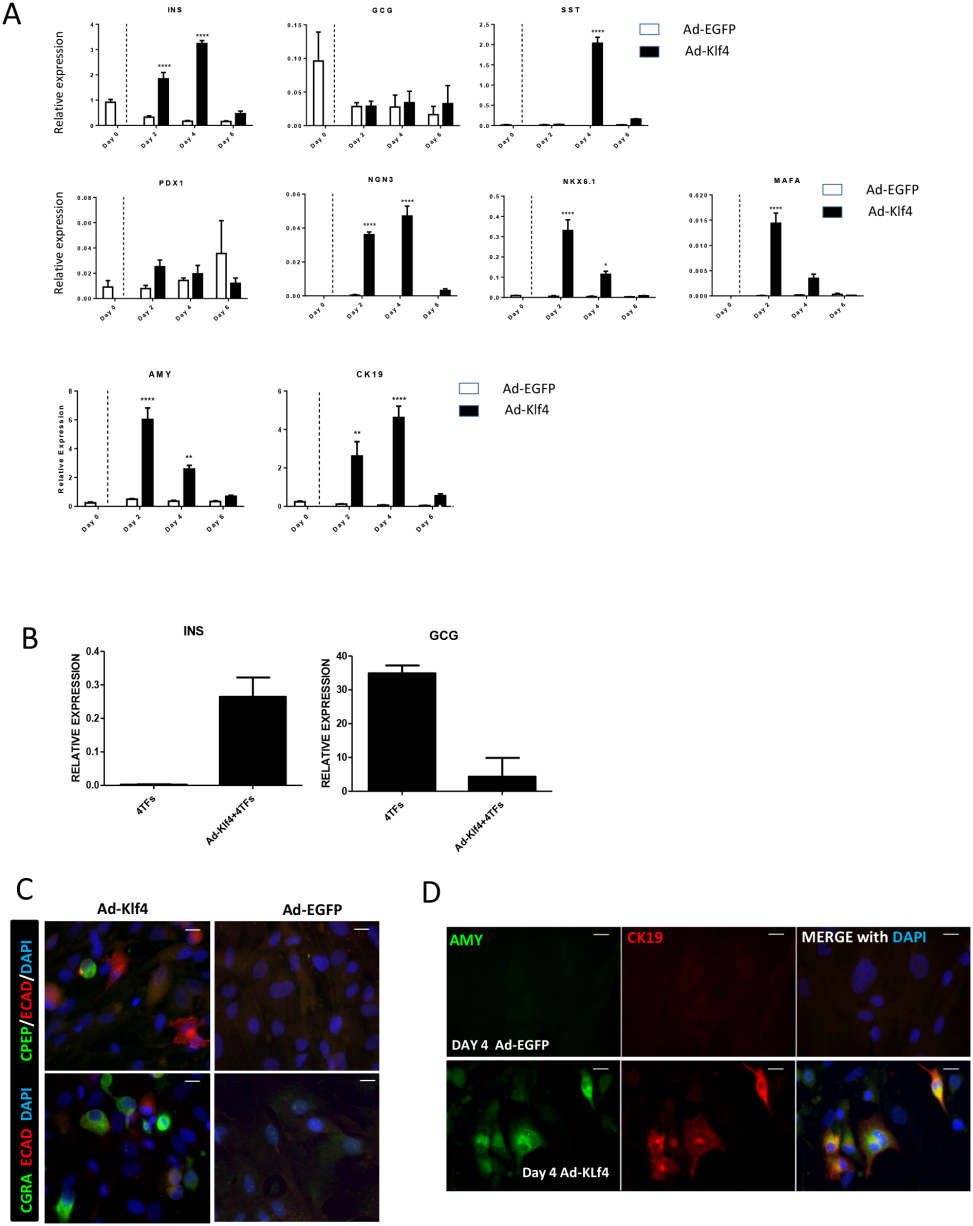
KLF4 overexpression induces re-expression of both endocrine and exocrine markers. Islet enriched pancreatic cell clusters were cultured in RPMI with 10% FBS and allowed to adhere and expand. At passage 6 cells were transduced with Ad-Klf4 or Ad-EGFP. (A) QRT/PCR analysis of pancreatic markers in transduced cells. The dotted line represents the time of adenoviral transduction. Data represent mean ± SEM (n = 3) (B) QRT/PCR analysis of insulin (INS) and glucagon (GCG) expression in cells transduced with Ad-Klf4 or Ad-Klf4 in combination with Ad-Pdx1, Ad-Ngn3, Ad-MafA and Ad-Pax4 (4TFs). Data are expressed relative to glyceraldehyde 3-phosphate dehydrogenase and expressed as mean ± SEM (n = 3). Samples transduced with Ad-Klf4 or Ad-EGFP were immunostained at day 4 for E-cadherin, C-peptide and Chromogranin A (C) and for Amylase and CK19 (D). Nuclei were counterstained with DAPI. Scale bar = 20μm.

In addition to the up-regulation of epithelial markers, Klf4 overexpression led to a significant transient increase in the expression of the endocrine hormones insulin and somatostatin (Fig 3A) and pancreatic transcription factors (PDX1, NGN3, NKX6.1 and MAFA) that are present in developing and mature β-cells (Fig 3A). Expression levels of the acinar marker amylase and ductal marker CK19 were also significantly increased (Fig 3B). Interestingly, there was no increase in expression of glucagon (Fig 3A). We have previously shown [11] that reprogramming of pancreatic MSC-like cells using Ad-Pdx1, Ad-MafA, Ad-Ngn3 and Ad-Pax4 (4TFs) generates functional glucagon expressing α-like cells with no detectable expression of insulin (Fig 3B). Suppression of EMT using TGFβ1 and Rho kinase inhibitors enhanced 4TF-mediated insulin expression and suppressed glucagon expression [11]. In keeping with this observation, a similar effect was observed when promoting MET with Ad-Klf4 in combination with the 4TFs (Fig 3B). The effect on insulin expression was transient, suggesting that 4TFs alone were unable to stabilise the transient effects of Klf4.

C-peptide positive cells were infrequently observed (10.4% ± 3.1%) by immunocytochemistry in Ad-Klf4 but not Ad-EGFP treated cells; however chromogranin A, a pan-endocrine marker was seen throughout (32.1% ± 2.2%) following Ad-Klf4 treatment (Fig 3C). Immunocytochemistry also revealed widespread staining for amylase (59.0% ± 12.13%) and CK19 (34.7% ± 5.6%) with many cells staining for both (Fig 3D). Similar co-expression of amylase and CK19 was observed during the dedifferentiation of exocrine enriched cells [11,26].

**Fig 4:**
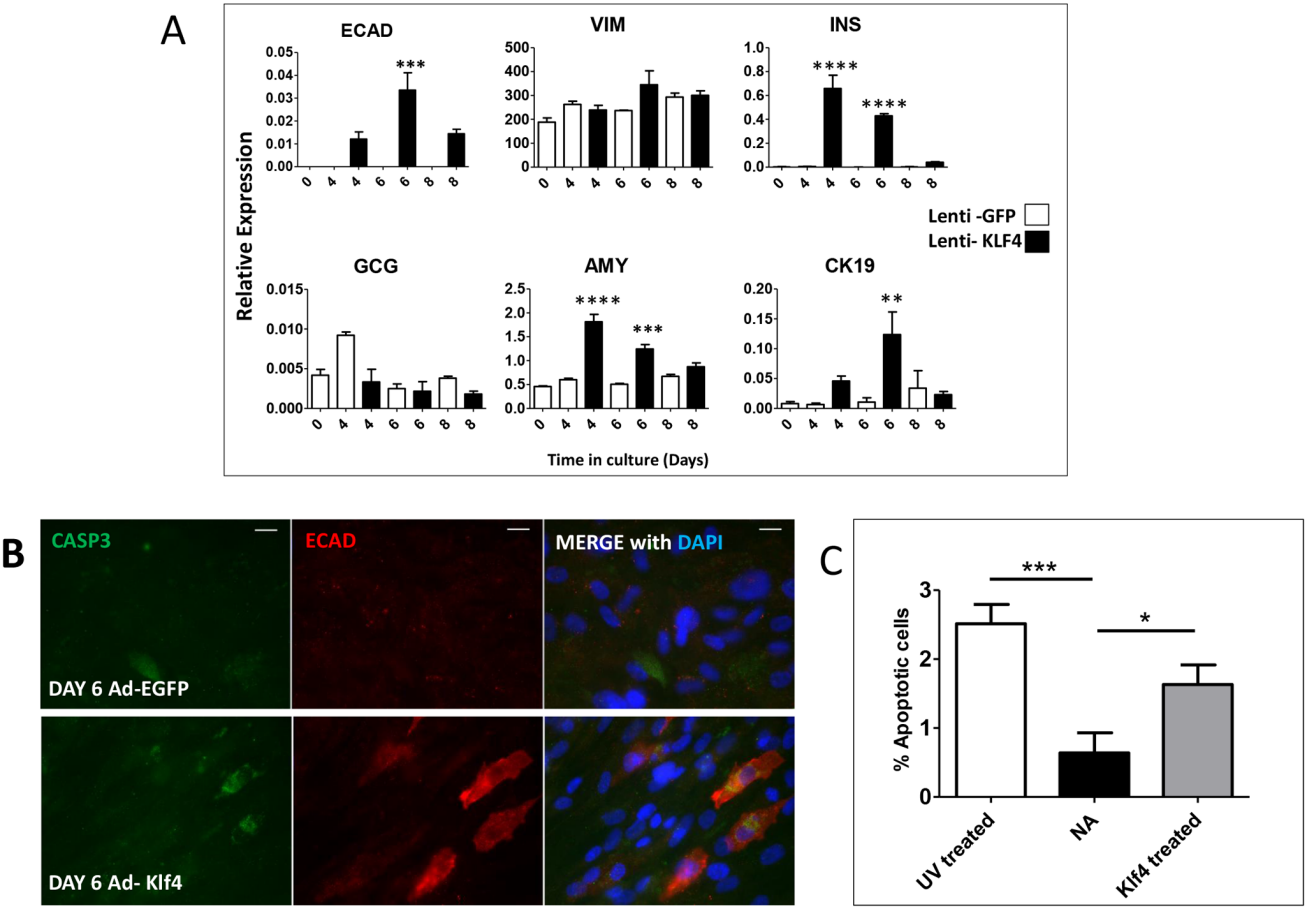
Effect of KLF4 is transient and induces apoptosis in treated cells. Islet-derived MSCs at passage 6 were transduced with lenti-KLF4 or lenti-EGFP and harvested at time points. (A) QRT/PCR analysis for expression of the indicated genes following culture for the indicated days after transduction with Lenti-Klf4 or Lenti-EGFP. The data are expressed relative to glyceraldehyde-3-phosphate dehydrogenase and represent the mean ± SEM (*n* = 3). A two-way ANOVA was performed on all QRT/PCR analyses with Bonferroni post hoc test comparing treatment groups with lenti-GFP. For all analyses, *P* *< 0.05 **P < 0.01 ***P < 0.001. (B) Cleaved caspase 3 (CASP3) co-expresses with E-cadherin (ECAD) in Ad-Klf4 but not Ad-EGFP treated cells. Nuclei were counterstained with DAPI. Scale bar = 20μm. (C) Cells were subjected to UV light or transduced with Ad-KLF4 or Ad-EGFP (NA) and fixed at day 4. A TUNEL assay was performed followed by counterstaining with DAPI. Over 1500 nuclei were counted per treatment and cells identified as apoptotic calculated as a percentage of all cells.

We hypothesised that the transient nature of the Klf4 effect could be attributed in part to the use of non-integrating adenoviral vectors. To address this we created a lentiviral vector overexpressing human KLF4, which would integrate into the host genome. Lenti-KLF4, but not Lenti-EGFP, induced an increase in E-cadherin, insulin, amylase and CK19, but not (as seen with Ad-Klf4) glucagon expression. However, as seen with the Ad-Klf4 construct, the increased expression of these markers was transient (Fig 4A). Collectively, these data suggest that exogenous Klf4 is capable of initiating a process of MET but that other factors might be required for further maturation and stabilisation of the epithelial phenotype. Some evidence in favour of the requirement for these factors was provided by the observed co-staining of the apoptotic marker CASP3 and E-cad in Ad-Klf4 infected cells (Fig 4B), while a TUNEL assay, which measured a later stage apoptosis, revealed a significantly higher number of apoptotic cells following treatment with Ad-Klf4 (Fig 4C).

### Promoting cell-to-cell contact in suspension culture enhances KLF4 induced redifferentiation

We next hypothesised that adjusting the cell culture environment to promote survival of newly formed epithelial cells would enhance redifferentiation. Initial experiments involved treatment with Ad-Klf4 along with the Rho-associated kinase inhibitor (ROCK) Y27632, which has previously been effective in preventing apoptosis in dissociated pluripotent stem cells [27] and suppressing pancreatic exocrine cell dedifferentiation [11]. However, no significant difference in gene expression was observed between treatment groups (data not shown). We next investigated whether coating the culture dish with different laminin isoforms, including those known to interact with β-cells in the human basal lamina [28], would enhance Ad-Klf4-mediated redifferentiation. Freshly-plated islet derived MSCs became fully attached to laminin isoforms LN511 and LN521 after only 8 hours, while attachment to other isoforms and to glass took significantly longer (a full 24 hours). Four days after Ad-Klf4 transduction, superior attachment was observed on the LN521 coating, but not on the other isoforms. However, none of the laminin isoforms enhanced Ad-Klf4 expression of insulin, amylase, CK19 and E-cadherin (data not shown).

**Fig 5:**
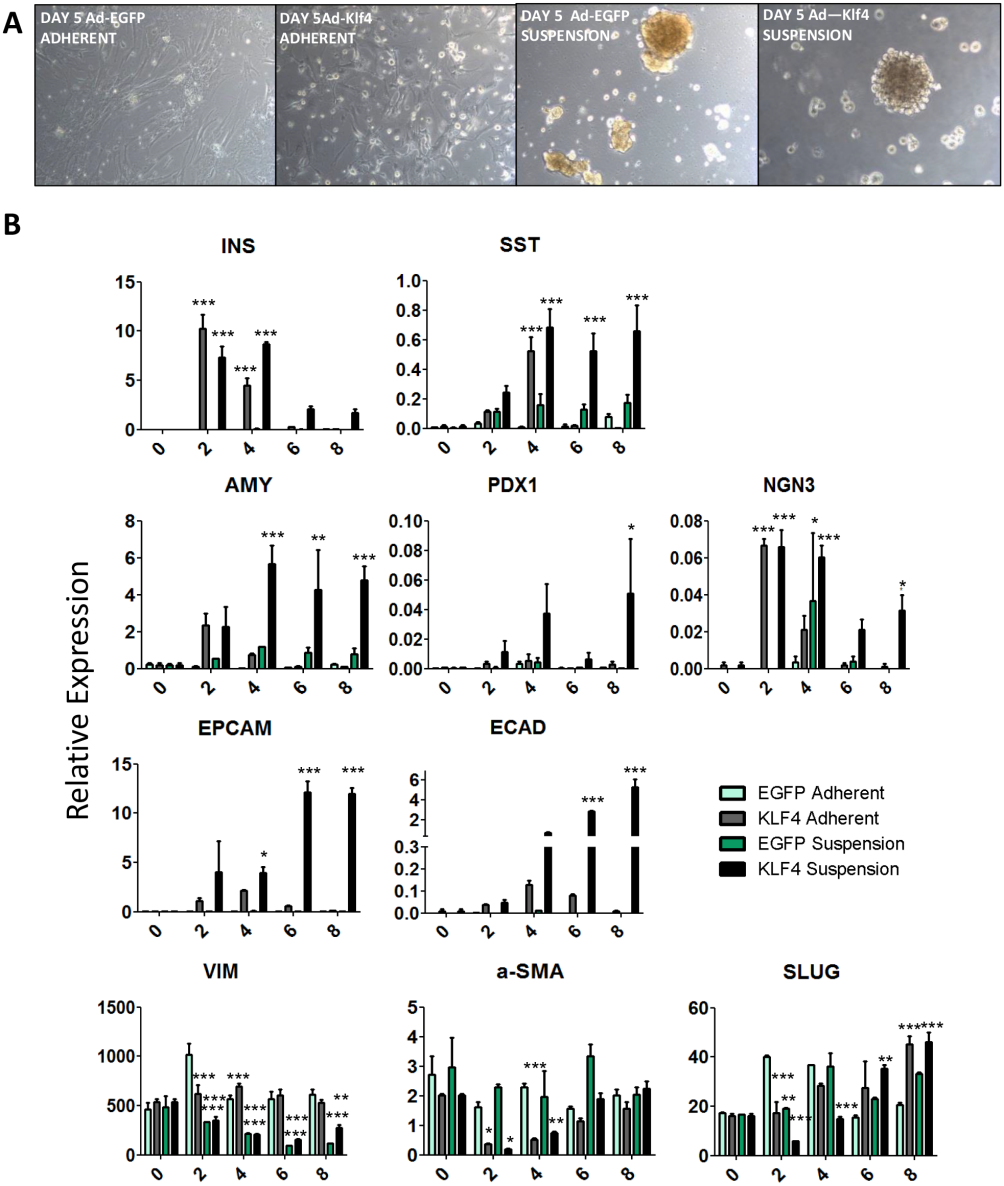
Suspension culture enhances the effects of Ad-Klf4. Islet-derived MSCs at passage 6 were transduced with Ad-Klf4 or Ad-EGFP and cultured overnight in adherent cell culture conditions. The cells were then either left in adherent conditions or transferred to suspension culture for a further 4 days. (A) Phase contrast comparison of cells in adherent or suspension culture at day 5. (B) Samples were harvested for QRT/PCR at several time points and analysed for expression of pancreatic, epithelial and mesenchymal markers. Data were expressed relative to glyceraldehyde-3-phosphate dehydrogenase and represent the mean ± SEM (*n* = 3). A one-way ANOVA was performed on all QRT/PCR analyses with Dunnett post hoc test comparing treatment groups with an Ad-EGFP control. Unpaired t-tests were performed where necessary. For all analyses, *P* *< 0.05 **P < 0.01 ***P < 0.001.

It has been previously shown that suspension culture in serum free media can enhance redifferentiation of islet- and exocrine-derived MSCs [5,29]. Culture in suspension for 5 days led to the formation of epithelial-like clusters (Fig 5A). In monolayer culture Ad-Klf4, but not Ad-EGFP, increased expression of epithelial markers, and this effect was considerably enhanced when the Ad-Klf4 treated cells were subsequently placed in suspension, under which conditions a marked decrease in mesenchymal markers (vimentin and SNAI2) was also observed (Fig 5B). However, the levels of the pancreatic markers insulin, amylase and Ngn3 decreased by day 6, with a concomitant increase in the mesenchymal markers α-SMA and SLUG, indicating that the effect of Ad-Klf4 remained transient.

### β-cell derived MSCs redifferentiate down both endocrine and exocrine lineages following treatment with Ad-KLF4

**Fig 6:**
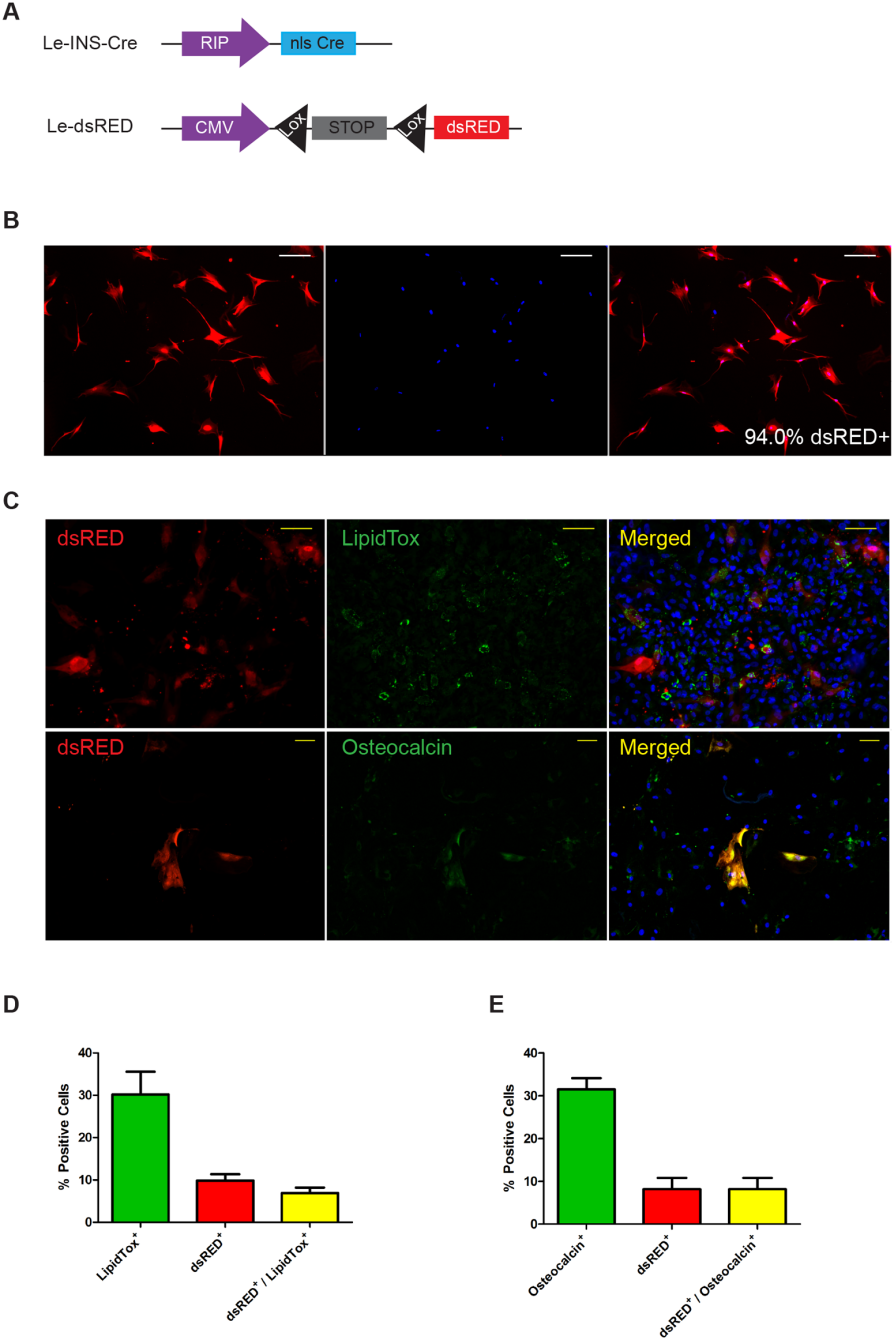
β-cell derived MSCs can be differentiated towards adipocyte and osteoblast lineages. (A) Islets were transduced 24h after plating with a lenti virus encoding Cre recombinase under the control of the insulin promoter (Le-INS-Cre) and a lentivirus encoding dsRED preceded by a floxed stop cassette (Le-dsRED). These lineage traced beta cells were allowed to expand in culture for 6 weeks. (B) The expanded dsRed^+^ beta-derived MSCs were FACS sorted and cultured up to passage 4. Left panel dsRed, middle DAPI, and right merged. (C) Immunostaining of sorted β-cell derived MSCs upon differentiation towards adipocytes (lipid droplets, LipidTox) or osteoblasts (osteocalcin). Scale bar = 50μm. Quantification of the percentage of differentiation towards adipocytes (D) and osteoblasts (E). >1500 nuclei were counted per treatment and cells identified as positive calculated as a percentage of total cells.

**Fig 7:**
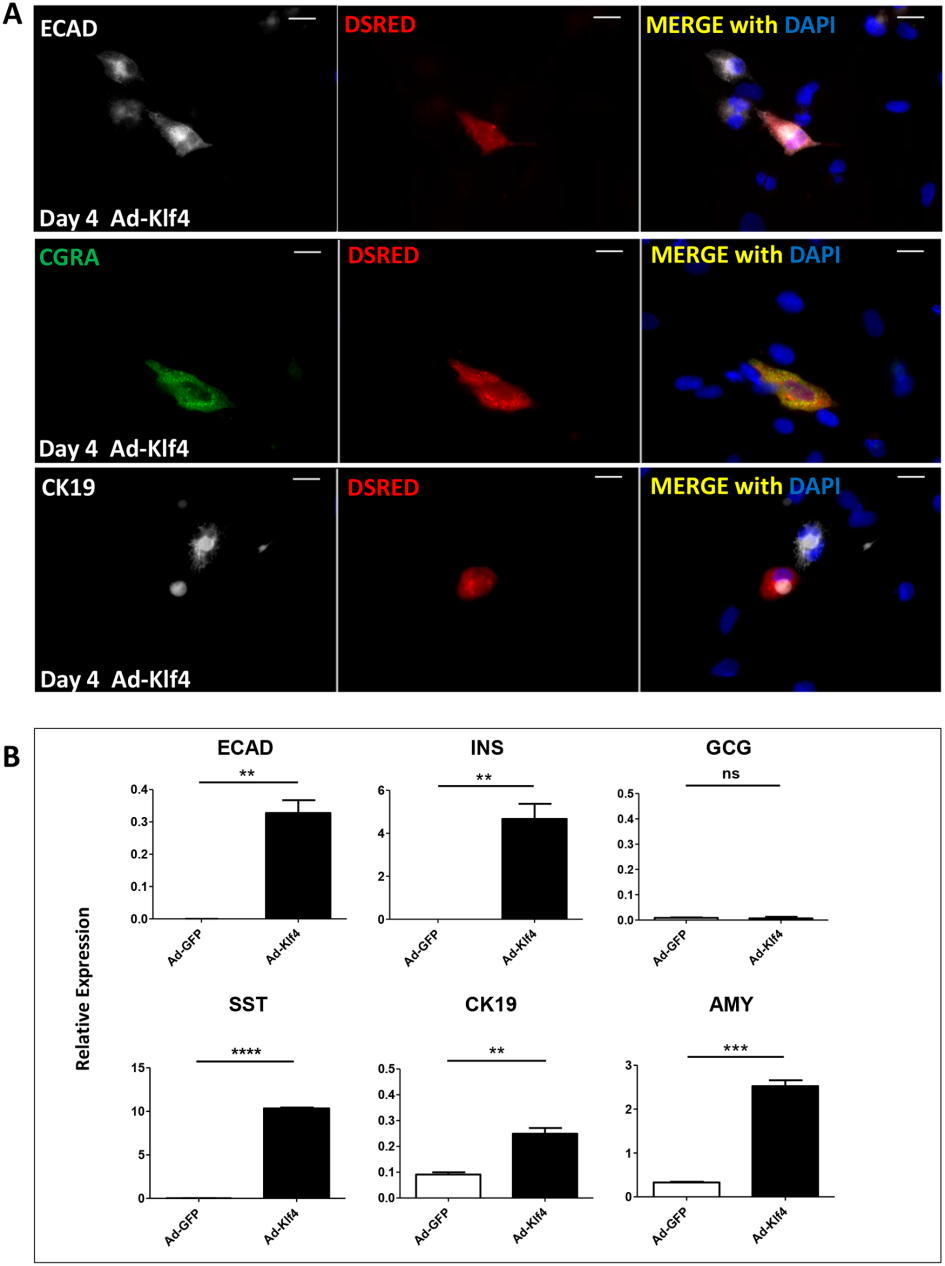
Ad-Klf4 induces INS-dsRed MSCs to differentiate down both endocrine and exocrine lineages. (A) FACS sorted and expanded INS-dsRED MSCs were transduced with Ad-Klf4, samples fixed for immunocytochemistry and stained for E-cadherin, chromogranin A and CK19. Nuclei were counterstained with DAPI. Scale bar = 20μm. (B) FACS sorted and expanded INS-dsRED MSCs were transduced with Ad-Klf4 or Ad-EGFP. Samples were harvested for QRT/PCR analysed for expression of epithelial and endocrine markers, relative to glyceraldehyde-3-phosphate dehydrogenase and represented as mean ± SEM (*n* = 3). Unpaired t-tests were performed between Ad-Klf4 and Ad-EGFP transduced cells. For all analyses, *P* *< 0.05 **P < 0.01 ***P < 0.001.

The question remained as to whether KLF4 was inducing a redifferentiation via MET of MSCs derived from dedifferentiated islet cells or was acting exclusively on an expanded endogenous stroma- derived population of MSCs. To investigate this we used a genetic lineage tracing approach that we have previously described [11,26]. This involved using a lentivirus containing Cre-recombinase under the control of the insulin promoter and a lentivirus containing a CMV driven dsRed reporter preceded by a floxed stop cassette blocking its expression (Fig 6A). The cells were allowed to dedifferentiate, and after several passages the dsRed positive cells were sorted by flow cytometry and expanded to provide almost homogeneous (>94%) populations of MSCs (Fig 6B) that were derived from insulin positive β-cells (INS-dsRed MSCs). We were then able to demonstrate that MSCs derived from β-cells could be induced to differentiate down adipocyte and osteoblast lineages (Fig 6C and 6D). When treated with Ad-Klf4 INS-dsRed cells were shown to co-stain for dsREd and E-cadherin (14.6% ± 2.6%), chromogranin A (14.3% ± 5.7%) and CK19 (6.7% ± 3.4%, Fig 7A), and by RT/QPCR to express E-cadherin, insulin, somatostatin, CK19 and amylase (but not glucagon) (Fig 7B). These effects were not seen with dsRed-enriched cells treated with Ad-EGFP. Collectively these results suggest that Ad-KLF4 can transiently reverse EMT in MSCs derived from β-cells. Similar results were obtained for AMY-dsRed MSCs [11], indicating that β-cell and acinar cell derived MSCs have the ability to redifferentiate towards both endocrine and exocrine lineages after long term culture.

## Discussion

We have previously shown that human exocrine-enriched cells can be efficiently reprogrammed into functional β-like cells, using a combination of four pancreatic transcription factors, namely Pdx1, MafA, Ngn3 and Pax4 [11]. Efficient reprogramming was dependent on EMT suppression through a combination of TGFβ1 and Rho-kinase inhibitors. When the exocrine cells were allowed to expand as an MSC population the same protocol generated, for reasons that are not completely understood, predominantly α-like cells. It was envisaged that the β-cell directed reprogramming protocol would provide a recipient-specific supply of new β-cells that could be used for subsequent transplants, since it often takes two and sometimes three transplants to attain insulin dependence, and as a top-up supply of β-cells as the initial graft deteriorated with time [30]. An alternative scenario, addressed here, was that a large supply of culture-expanded allogeneic MSCs from a single donor could be redifferentiated and used to treat a large number of diabetic patients. The aim of this study was therefore to discover mechanisms that would reverse the dedifferentiation process that pancreatic islet tissue undergoes when placed in culture. There are very few proven strategies to induce MET in culture. Because of its role in inducing MET during the generation of iPS cells, we hypothesised that KLF4 might also act as an inducer of MET in MSCs expanded from human islets.

The major finding of the study was that adenovirus mediated overexpression of Klf4 in MSCs derived from islet enriched pancreatic cells drives MET and redifferentiation towards pancreatic cell types. Recent studies provide insights into how Klf4 might be acting in this process [31]. Klf4 belongs to a family of pioneer transcription factors, which can bind to highly condensed heterochromatin [32]. Through mechanisms that are not completely understood binding of Klf4 allows the chromatin to “breathe” thus allowing binding of other transcription factors to initiate gene transcription [33]. The three of the four transcription factors used in the reprogramming of iPSCs, i.e. Oct4, Sox2 and Klf4 (OSK) are all pioneer factors [19]. In our studies KLf4 was shown to reverse the EMT that pancreatic cells underwent in culture; however this was associated with increased apoptosis in Klf4- expressing cells. This suggests that Klf4 can make accessible not only sites within silent chromatin that control MET, but also additional sites, including those responsible for initiating an apoptotic cascade. Such a process may also occur during the generation of iPSCs and in part explain the very low efficiency of iPSC production.

Culture-expanded MSCs consist of a heterogeneous population of cells exhibiting a spectrum of phenotypes and functional properties [34]. The extent of this heterogeneity is dependent on a number of variables including: the tissue, donor and species of origin, isolation technique, culturing protocols, media used, and passage number [35,36]. We show here that MSCs derived from insulin positive β-cells or amylase-positive acinar cells are functionally equivalent, in that they can both be induced by Ad-Klf4 to express endocrine and exocrine markers and have the feature common to all MSC populations of being able to differentiate towards adipose and osteogenic lineages. This has important implications for cell therapy approaches to the treatment of type 1 diabetes since dedifferentiation, expansion, and redifferentiation of pancreatic tissue left over from the islet isolation procedure could provide a potentially unlimited supply of islets for transplantation [2]. The current study demonstrates that islet isolates that were not able to be utilised due to not meeting adequate numbers (<200, 000 islet equivalent units), as well as islet tissue left over from isolates that have been directed towards transplantation, or indeed exocrine tissue, would be equally useful for this purpose.

Overall the results suggest that Klf4 may play a pivotal role in attempts to generate an expandable source of islets for transplantation. However, further experiments should focus on minimising the off-target effects of Klf4 that result in apoptosis. This could be achieved by: 1. combining Klf4 with modified reprogramming protocols [11]; 2. altering the structure of Klf4; and 3. screening libraries containing DNA sequences that contain the required functional properties of Klf4 (and potentially other pioneer factors).
